# Phenotypic and Molecular Characterization of *Staphylococcus aureus* in Dairy Farms from Henan Province and the Inner Mongolia Autonomous Region, China

**DOI:** 10.3390/microorganisms12112150

**Published:** 2024-10-25

**Authors:** Mingquan Cui, Hejia Wang, Zekun Li, Ningning Han, Jie Li, Wenxiu Peng, Xiuying Zhang, Qi Zhao, Xuan Wang

**Affiliations:** 1 China Institute of Veterinary Drug Control, Beijing 100081, China; 2 Key Laboratory of Animal Antimicrobial Resistance Surveillance, Ministry of Agriculture and Rural Affairs, Beijing 100081, China

**Keywords:** biofilm formation, antimicrobial resistance, virulence, *S. aureus*, bulk tank milk

## Abstract

*Staphylococcus aureus*, a prevalent pathogen associated with infectious and foodborne diseases, is also a significant cause of intramammary infections in dairy farms. This study aimed to determine the phenotypic and molecular characterization of *S. aureus* in two different stock sizes of dairy farms in Henan province (HN) and the Inner Mongolia autonomous region (IM), China, through biofilm formation, antimicrobial resistance, virulence, and molecular type of *S. aureus* isolates. In HN, 74 *S. aureus* isolates (60.7%) were recovered from 122 bulk tank milk samples, while in IM, 24 *S. aureus* isolates (17.4%) were detected from 161 samples soured from various origins. Notably, 25.7% (19/74) of isolates in HN and 20.8% (5/24) in IM exhibited multidrug-resistant (MDR) phenotypes. Molecular typing revealed distinct patterns: ST97 (n = 32) and *spa* type t189 (n = 20) predominated in HN, whereas ST50 (n = 13) and *spa* type t518 (n = 11) were prevalent in IM. Additionally, three isolates harbored both *tsst-1* and *lukF-PV* genes, and two MRSA strains displayed a MDR phenotype in raw milk samples from HN. Biofilm formation was observed in 91.8% strains. Phylogenetic analysis identified two subpopulations (lineages 1 and 2). Among them, cluster 6 in lineage 2 comprised *S. aureus* strains from three sources within a farm, suggesting potential cross contamination during different stages in IM. Remarkably, among 19 MDR isolates in HN, ST398 MSSA strains exhibited a higher multidrug resistance compared to non-ST398 MSSA strains. This study underscores the high prevalence and diverse characteristics of *S. aureus* in raw milk, necessitating enhanced surveillance and control measures to mitigate associated risk.

## 1. Introduction

*Staphylococcus aureus*, a leading cause of bovine mastitis and human foodborne illnesses, has garnered significant attention due to its increasing antibiotic resistance [1]. Antibiotic therapy is the primary treatment for *S. aureus* infections. However, the emergence of antibiotic-resistant strains, particularly methicillin-resistant *S. aureus* (MRSA), poses a global challenge. MRSA, classified as a high-priority pathogen by the World Health Organization (WHO), is especially problematic in clinical settings due to its multiple antibiotic resistance [2]. The pathogenesis of *S. aureus* infections is attributed to various virulence factors. Representatively, toxic shock syndrome toxin-1 (TSST-1), a typical superantigen, induces toxic shock syndrome [3]; Panton–Valentine leucocidin (PVL) is a pore-forming toxin secreted by *S. aureus* that has recently been associated with necrotizing pneumonia [4]; Von Willebrand factor-binding protein (vWbp) is a hysteretic conformational activator, and interacts with prothrombin, leading to the conversion of fibrinogen to fibrin [5]. Furthermore, *S. aureus* can carry antimicrobial resistance (AMR) genes, including erythromycin resistance genes *erm(B)* and *erm(C)*, penicillin resistance gene *blaZ*, chloramphenicol resistance gene *cat*, lincomycin resistance gene *lnu(A)*, methicillin resistance gene *mecA*, and tetracycline resistance gene *tet(K)*, which reduce the effectiveness of the antimicrobial treatment of *S. aureus* mastitis [6]. Moreover, the ability of *S. aureus* to form biofilms contributes significantly to its persistence in the stressful environment. Research has focus on the virulence characterization and diversity of *S. aureus* in dairy farms, which serves as the starting point of the milk production chain. Previous studies have highlighted the potential risk of raw milk-derived MRSA ST398 infections in humans in China [7,8]. However, there is a lack of information regarding the characterization of MRSA or *S. aureus* strains in major milk production areas such as Henan province (HN) and the Inner Mongolia autonomous region (IM) in China. Given recent reports of increasing AMR and virulence in *S. aureus* isolated from raw milk [7,8], it is imperative to investigate the prevalence of *S. aureus* in this raw milk from major milk production areas in China. Hence, this study aimed to conduct a thorough examination of the prevalence, biofilm formation, AMR, virulence, and molecular type of *S. aureus* from dairy farms located in HN and IM, China.

## 2. Materials and Methods

### 2.1. Sample Collection

A total of 283 samples (detailed in Table 1) were collected from 109 non-large farms with an average stock size of less than 1000 in HN and 9 large dairy farms with stock sizes of over 1000 cows in IM during 2020. A total of 122 bulk tank milk samples were collected from 109 dairy farms in HN. Of these samples, two samples were collected for each farm for 13 large dairy farms, while one sample was collected for each farm for the other relatively small dairy farms. Additionally, 161 samples, including raw milk, mastitis milk, and nose swabs for workers and mastitis dairy cow, were gathered from multiple geographical areas in IM. Multiple samples were collected from bulk tanks, mastitis dairy cows, and workers to investigate the potential transmission risk within the farm. The geographical location of the sampling area is shown in Supplementary Figure S1.

For milk samples, 25–40 mL milk was collected in a 50 mL sterile centrifuge tube from a milk bulk tank in each dairy farm. Nasal swabs were taken from individual workers and mastitis dairy cows within the farms and were then transferred to a 15 mL centrifuge tube containing 10 mL of 10% sodium chloride tryptic soy broth. All samples obtained were promptly transported to the laboratory in an insulated ice chest containing ice packs, and microbial analysis was performed immediately upon arrival at the laboratory.

### 2.2. Isolation of S. aureus

The 1 mL of raw milk sample or one nose swab was mixed with 10 mL of 10% sodium chloride broth, and the mixture was incubated overnight at 37 °C for enrichment. An aliquot of the bacterial suspension was then transferred uniformly onto CHRO Magar *S. aureus* isolation medium (CHRO Magar, Paris, France) followed by incubation for 24 h at 37 °C. One colony was then selected and inoculated in 1 mL of brain heart infusion (BHI) (Land Bridge, Beijing, China) for incubation for 6 h at 37 °C under shaking. The identification of selected colonies as *S. aureus* was conducted using a Vitek 2 automated microbial identification system [9].

### 2.3. Antimicrobial Susceptibility Testing of S. aureus

Susceptibilities to nine antimicrobials—penicillin, oxacillin, cefoxitin, gentamicin, trimethoprim/sulfamethoxazole, sulfisoxazole, clindamycin, erythromycin, and ofloxacin—were determined using the broth dilution method. The results were interpreted according to the Clinical and Laboratory Standards Institute (CLSI) guidelines [10]. *S. aureus* ATCC 29213 was used in assays as the quality control strain. *S. aureus* isolates was considered multidrug resistant (MDR) when they showed resistance to three or more antimicrobial classes of antimicrobial agents [11].

### 2.4. Biofilm Formation Assays

Biofilm production by *S. aureus* strains grown in trypticase soy broth (TSB) was determined using a semi-quantitative adherence assay on 96-well tissue culture plates as described previously [12]. Briefly, *S. aureus* isolates were placed in test tubes containing TSB, incubated at 37 °C for 24 h, and diluted to 10^8^ CFU/mL. Then, 2 μL of the diluted culture was added to 198 μL TSB (1:100) in a sterile 96-well culture plate. A 200 μL aliquot of TSB was used as the negative control. The biofilm was formed by 37 °C incubation for 24 h. Then, the plate was removed and gently washed with 200 μL PBS. Subsequently, 200 μL of methanol was added to fix the formed biofilm for 20 min, and then 150 μL of 1% crystal violet (CV) was used to dye the biofilm for 15 min. The wells were washed three times in PBS to remove the excess dye. The microplate was air-dried, and the biofilm combined with CV was dissolved in 150 μL of a mixture of absolute ethanol and acetone with 80:20 volume ratio. The optical density (OD_570_) of each strain was measured in a microplate reader by the arithmetic mean of the absorbance of three wells at 570 nm, then this value was compared with the mean absorbance of negative controls (ODc). The following classification was used for the determination of biofilm formation: no biofilm production (OD_570_ ≤ ODc), weak biofilm production (ODc < OD_570_ ≤ 2 × ODc), moderate biofilm production (2 × ODc < OD_570_ ≤ 4 × ODc) and strong biofilm production (4 × ODc ≤ OD_570_).

### 2.5. Whole-Genome Sequencing (WGS) and Bioinformatics

The genomic DNA of all strains was extracted from overnight cultures grown at 37 °C using a TIANamp Bacteria DNA Reagent Kit (Tiangen, Beijing, China) according to the manufacturer’s instructions. DNA was used as the template for the construction of indexed DNA libraries, and whole-genome sequencing was conducted using the Illumina HiSeq 2500 system [13]. Obtained raw data were assembled using SPAdes Genome Assembler (v3.9.0) [14], and assembled genome data were submitted to the NCBI database (PRJNA1103959). Multilocus sequence typing (MLST), AMR and virulence genes were identified using ABRicate [15,16], staphylococcal cassette chromosome *mec* (SCC*mec*) and *spa* types were identified using available pipelines (http://www.genomicepidemiology.org// accessed on 23 June 2021). To elucidate the evolutionary relationships of *S. aureus* isolates, a neighbor-joining phylogenetic tree was conducted using snippy soft (v4.6.0) (https://github.com/tseemann/snippy/ accessed on 25 June 2021).

### 2.6. Ethical Approval

This study was conducted in accordance with the Declaration of Helsinki, and the protocol was approved by the Ethics Committee of China Institute of Veterinary Drug Control (approval no. 202000077) on 15 April 2020. The oral permissions were obtained from the farm workers regarding milk and nose collection.

## 3. Results and Discussion

### 3.1. Isolation, Identification and Susceptibility Profiles of S. aureus

The prevalence of *S. aureus* isolates from two regions in China is summarized in Table 1. A total of 74 *S. aureus* isolates (60.7%) were recovered from 122 raw milk samples from a bulk tank in HN, and 16 *S. aureus* isolates (48.5%) were recovered from 33 raw milk samples in IM. However, the isolation rate was lower in mastitis milk samples, with only 6 *S. aureus* strains (7.5%) isolated from 80 samples. Compared to the isolation rate, the prevalence of *S. aureus* in mastitis milk was recorded as 46.2%, 28.5%, and 30.3% in Beijing, Italy, and Pakistan [17,18,19]. Furthermore, two *S. aureus* isolates (5.6%) were detected among 36 nose swabs collected from mastitis dairy cows, while no *S. aureus* strain was found in 12 nose swabs from farm workers. Although the isolation of *S. aureus* from milk and dairy products is not uncommon [20], the detection rate observed in this study is higher compared to rates (detailed in Supplementary Table S1) reported in raw milk from ten provinces across China, Japan, and Bangladesh [7,8,21,22], indicating that the presence of *S. aureus* in raw milk deserves significant concern, regardless of whether the milk comes from large or non-large dairy farms.

All 98 *S. aureus* strains were tested for their susceptibility to 9 antimicrobial agents, and the resistance is summarized in terms of sample sources in Table 2. Alarmingly, 98 (100%) *S. aureus* isolates were resistant to penicillin. The high resistance rate to penicillin was similar to that of raw milk-derived *S. aureus* isolates from Xinjiang, suggesting that penicillin should be used cautiously for mastitis caused by *S. aureus* [23]. The resistance rate to the other antimicrobial agents was relatively low (<50%). Notably, a high resistance rate (≥50%) was observed for ofloxacin and sulfaisoxazole among *S. aureus* isolates from mastitis cow samples. A total of 24 strains showed MDR phenotypes.Among them, 25.7% (19/74) *S. aureus* strains in HN and 20.8% (5/24) in IM exhibited MDR phenotypes. Intriguingly, only two *mecA*-positive MRSA strains, exhibiting two distinct MDR phenotype patterns, were identified in raw milk samples from two cities in HN. All other *S. aureus* isolates were identified as methicillin-sensitive *S. aureus* (MSSA). One MRSA isolate (HN-37) showed resistance to penicillin, cefoxitin, oxacillin, erythromycin, clindamycin, and sulfaisoxzole. The other MRSA isolate (HN-114) was resistant to penicillin, erythromycin, and clindamycin but sensitive to cefoxitin (MIC, 1 μg/mL) and oxacillin (MIC, 0.25 μg/mL). It is noteworthy that oxacillin-susceptible *mecA*-positive (OS-MRSA) isolates have been described in raw milk, retail food [24] and humans [25] in China.

### 3.2. Biofilm Formation of S. aureus

**Figure 1:**
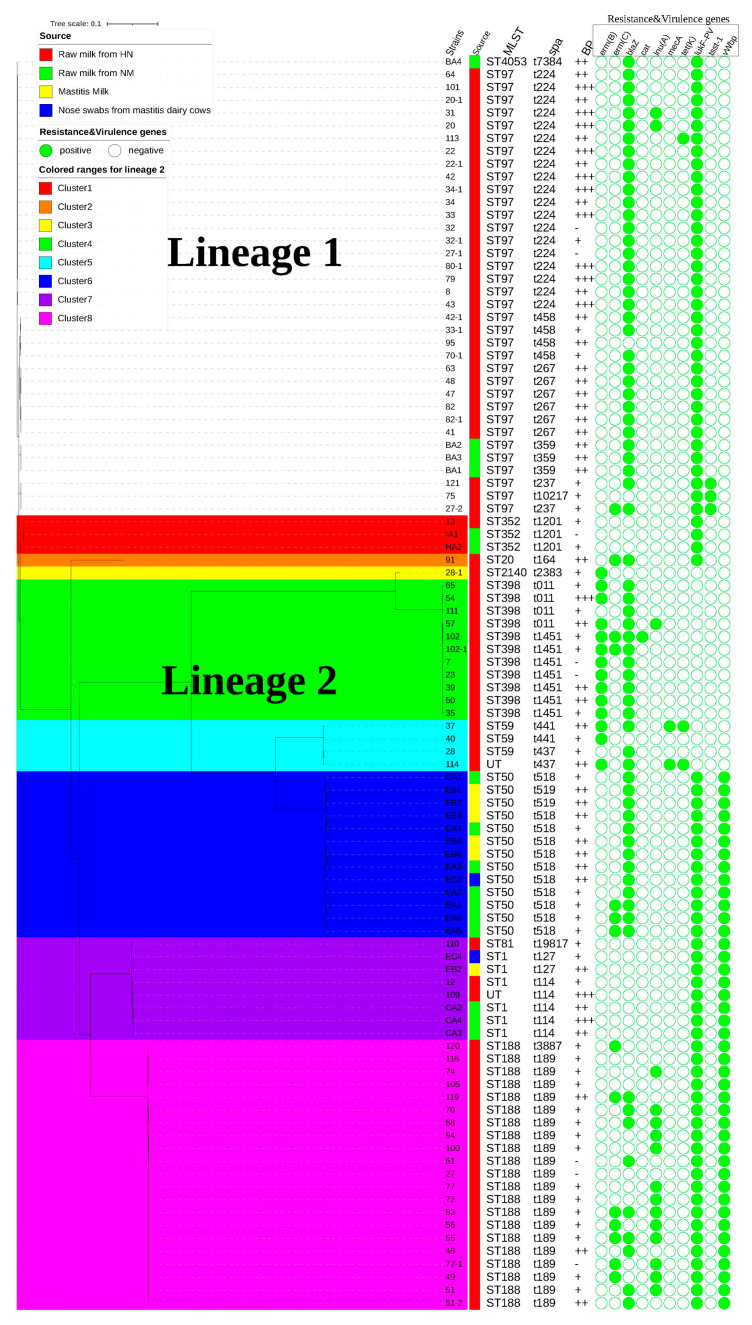
Phylogenetic tree of 98 *S.aureus* isolates from dairy farms in HN and IM. MLST: UT indicates untypeable; BP (biofilm production): − means no biofilm production, + means weak biofilm production, ++ means moderate biofilm production, and +++ means strong biofilm production.

The biofilm production capacity of 98 *S. aureus* strains was evaluated, and the results are presented in Figure 1 and summarized in Table 2. A recent report indicated that most *S. aureus* strains possess biofilm formation ability, and the adversity resistance associated with biofilm formation is more obvious in the milk-isolated strains compared with human-derived strains [26]. In our study, only eight *S. aureus* strains were biofilm-negative producers, whereas most *S. aureus* strains (91.8%, 90/98) exhibited biofilm formation ability in the dairy farms as summarized in Table 2. A previous study pointed out that all *S. aureus* isolates could form biofilms, and at least 80% of isolates showed the ability to produce strong biofilms [7]. In this study, most *S. aureus* strains were similarly able to form biofilms, but the proportion of *S. aureus* strains forming strong biofilms was low. Among them, two raw milk-derived MRSA isolates displayed moderate biofilm formation, and twelve *S. aureus* strains (16.2%, 12/74) from HN and one strain (4.2%, 1/24) from IM were classified as strong biofilm producers in a bulk tank milk. Biofilm not only enables *S. aureus* to tolerate poor environments, but also reduces the effects of antimicrobial agents, increasing the difficulty of elimination from equipment. Therefore, in this study, we speculated that the widespread biofilm formation capacity of *S. aureus* might be one of the important reasons for the widespread prevalence of *S. aureus* in the dairy farm. Particularly, the enhanced biofilm formation capacity may contribute to the persistence of the microorganism during food processing, thereby increasing the risk of food contamination and posing a potential threat to consumers’ health [27].

### 3.3. Antimicrobial Resistance and Virulence Genes, Molecular and Phylogenetic Analysis of S. aureus

The phylogenetic analysis revealed the classification of the 98 strains into two distinct subpopulations, designated as lineages 1 and 2. Notably, 36 strains isolated from a bulk tank milk in HN and IM belonged to lineage 1, while the remaining 62 strains were grouped into lineage 2, further categorized into clusters 1–8. Most *S. aureus* strains from IM clustered within clusters 6 and 7, whereas those from HN were primarily distributed across the other clusters. Interestingly, cluster 6 mainly involved the *S. aureus* strains sourced from three distinct locations (strains with prefix named EA from a bulk tank, EB from mastitis milk, EC from nose swabs from mastitis dairy cow) within the same dairy farm, suggesting the possibility of cross contamination of *S. aureus* strains across different stages. Compared to previous studies on the phenotype and molecular characterization of *S. aureus* samples from most dairy farms, most studies only focused on healthy cows or mastitis cows [8,19,28]. To our knowledge, this study was the first attempt to simultaneously correlate healthy cows and mastitis cows from dairy farms for the phenotype and molecular characterization of *S. aureus*.

The AMR genes *erm(B)*, *erm(C)*, *blaZ*, *cat*, *lnu(A)*, *tet(K)*, and *mecA* and virulence genes *lukF-PV*, *tsst-1*, and *vWbp* were screened in all 98 *S. aureus* strains and the results are highlighted in Figure 1. Among them, *blaZ* and *lukF-PV* were widely present in *S. aureus* strains from two regions, which was similar to the high detection rate of *S. aureus* carrying *blaZ* and *lukF-PV* genes in Egypt [29], while *vwbp* accompanied with *lukF-PV* was widely present in *S. aureus* strains from IM. The remaining genes were observed sporadically within *S. aureus* strains sourced from two regions. Our findings indicated that the most prevalent resistance gene was penicillin resistance gene *blaZ*, which showed that the resistant phenotypes and genotypes of *S. aureus* were not completely consistent. One possible explanation for this finding is that phenotypic resistance may not be caused by genetic acquisition, or the intrinsic mechanisms of bacterial resistance (such as biofilms or multidrug efflux pumps) may play a primary role in resistance [30]. Notably, all *S. aureus* isolates within clusters 3–5, except for strains 28 and 111, possessed the *erm(B)* gene, while all *S. aureus* strains in these clusters demonstrated the absence of the *lukF-PV* gene. Furthermore, we identified three strains (121, 75 and 27-2) within lineage 1 that unexpectedly harbored both the *tsst-1* and *lukF-PV* genes. It is rare for a single strain of *S. aureus* to possess both these simultaneously, as their coexistence is associated with severe human *S. aureus* infections [31]. Similarly, the evidence for *tsst-1* carried by raw milk-derived *S. aureus* in this study has been reported in Portugal and Poland [32,33].

The results regarding MLST STs and *spa* typing are presented in Figure 1. Among 74 bulk tank milk-derived *S. aureus* isolates in HN, a range of STs were identified, with ST97 being the most prevalent (n = 32), followed by ST188 (n = 21), and ST398 (n = 11). Other STs included ST59 (n = 3), ST2140 (n = 3), ST1 (n = 1), ST352 (n = 1), ST20 (n = 1), and ST81 (n = 1). Two isolates remained untypable. However, ST50 (n = 7) was frequently identified in bulk tank milk-derived *S. aureus* in IM. Additionally, ST97 (n = 3), ST1 (n = 3), ST352 (n = 2) and ST4053 (n = 1) were also observed. In mastitis cow-derived *S. aureus*, ST50 (n = 6) and ST1 (n = 2) were detected. It is worth pointing out that ST97 molecular typing is widely present in European countries for both raw milk and mastitis milk-derived *S. aureus*, while ST50 has only been reported in Germany for milk-derived *S. aureus* [28,34]. Notably, all five *S. aureus* strains with a MDR phenotype in IM were typed ST50, and originated from bulk tank milk (n = 3) and mastitis-derived milk (n = 2). Among 19 *S. aureus* strains with a MDR phenotype in HN, ST398 was the most prevalent (n = 10), followed by ST97 (n = 4), ST59 (n = 3), ST20 (n = 1), and one untypable isolate. These findings were similar to the previous reports indicating that MSSA ST398 strains exhibited higher levels of multidrug resistance phenotypes compared to MSSA non-ST398 strains [35,36]. Figure 1 also provides detailed information on *spa* types, revealing that t189 (n = 20) and t224 (n = 19) were commonly detected among 74 *S. aureus* isolates from HN, while t518 (n = 11) was frequently detected among the 24 *S. aureus* isolates from IM. Additionally, two *mecA*-positive MRSA strains (HN-37 and HN-114) were identified as ST59-t441-SCC*mec* IV and t437-SCC*mec* V, respectively.

## 4. Conclusions

Previously, we extensively studied the molecular epidemiological characteristics of MRSA, particularly MRSA ST398, in raw milk in China. This study serves as an important supplement to research on MRSA isolated from dairy farms across various regions in China. Here, epidemiological investigation of these *S. aureus* strains was undertaken by comprehensive analyses incorporating genotypic and phenotypic data to examine the prevalence, antimicrobial resistance, AMR and virulence genes, biofilm formation, molecular type, and phylogenetic relation. These data provide comprehensive insights into *S. aureus* prevalence in raw milk from two significant milk production areas. These specific clinical properties, such as a high resistance rate to penicillin, extensive biofilm formation, rare simultaneous carriage of *tsst-1* and *lukF-PV* virulence genes, and the MDR phenotypes exhibited by MSSA ST398, posed significant challenges to the clinical treatment of bovine mastitis caused by *S. aureus*, while also posing a major threat to human food hygiene. Therefore, it is necessary to strengthen the monitoring of the prevalence of *S. aureus* in dairy farms, and improve sanitation measures to thwart biofilm development in dairy farms.

## Figures and Tables

**Table 1:** Prevalence of *S. aureus* and MRSA from different resources in Henan Province and the Inner Mongolia autonomous region.

Regions ^a^	Source	Dairy Farms	Total No. of Sample	No. of *S. aureus* Isolates	No. of MRSA Isolates
HN	Raw milk from bulk tank	109	122	74	2
IM	Raw milk from bulk tank	9	33	16	0
	Mastitis Milk	9	80	6	0
	Nose swabs from mastitis dairy cows	5	36	2	0
	Nose swabs from farm workers	5	12	0	0

^a^ HN, Henan province; IM, Inner Mongolia autonomous region.

**Table 2:** The phenotypes for antimicrobial resistance and biofilm formation of *S. aureus* from different resources in Henan Province and the Inner Mongolia autonomous region.

Regions ^a^	Source (No. of Isolates)	No. (%) of Isolates Resistant to Drug ^b^									No. (%) of Isolates Classified by Biofilm Production ^c^			
		PEN	ERY	CLI	OFL	FOX	SF	OXA	SXT	GEN	No	Weak	Moderate	Strong
HN	Raw milk from bulk tank (n = 74)	74 (100)	22 (29.7)	10 (13.5)	16 (21.6)	1 (1.4)	32 (43.2)	1 (1.4)	14 (18.9)	7 (9.5)	7 (9.5)	32 (43.2)	23 (31.1)	12 (16.2)
IM	Raw milk from bulk tank (n = 16)	16 (100)	3 (18.8)	3 (18.8)	7 (43.8)	0	9 (56.3)	0	0	0	1 (6.3)	7 (43.8)	7 (43.8)	1 (6.3)
	Mastitis Milk (n = 6)	6 (100)	0	0	5 (83.3)	0	3 (50.0)	0	0	0	0	0	6 (100)	0
	Nose swabs from mastitis cows (n = 2)	2 (100)	0	0	1 (50.0)	0	1 (50.0)	0	0	0	0	1 (50)	1 (50)	0
Total		98 (100)	25 (25.5)	13 (13.3)	29 (29.6)	1 (1)	45 (45.9)	1 (1)	14 (14.3)	7 (7.1)	8 (8.2)	40 (40.8)	37 (37.8)	13 (13.3)

^a^ HN, Henan province; IM, Inner Mongolia autonomous region. ^b^ PEN, penicillin; ERY, erythromycin; CLI, clindamycin; OFL, ofloxacin; FOX, cefoxitin; SF, sulfaisoxazole; OXA, oxacillin; SXT, trimethoprim/sulfamethoxazole; GEN, gentamicin. ^c^ No, no biofilm production; Weak, weak biofilm production; Moderate, moderate biofilm production; Strong, strong biofilm production.

## Data Availability

The original contributions presented in this study are included in this article/Supplementary Material. Further inquiries can be directed to the corresponding author.

## Supplementary Materials

The following supporting information can be downloaded at: https://www.mdpi.com/article/10.3390/microorganisms12112150/s1. Figure S1. Geographical location of dairy farms presenting *S. aureus* and MRSA isolates in Henan province (HN) and the Inner Mongolia autonomous region (IM), China; Table S1. Comparison of the prevalence of raw milk-derived *S. aureus* and MRSA in different regions.
