# Supplementary material for: Phenotypic and Molecular Characterization of Staphylococcus aureus in Dairy Farms from Henan Province and the Inner Mongolia Autonomous Region, China
Source: Microorganisms. 2024 Oct 25;12(11):2150. doi: 10.3390/microorganisms12112150 (PMC11596393; doi:10.3390/microorganisms12112150)
Supplement: Supplementary file 1 [file microorganisms-12-02150-s001.zip › microorganisms-3266878-supplementary.pdf]

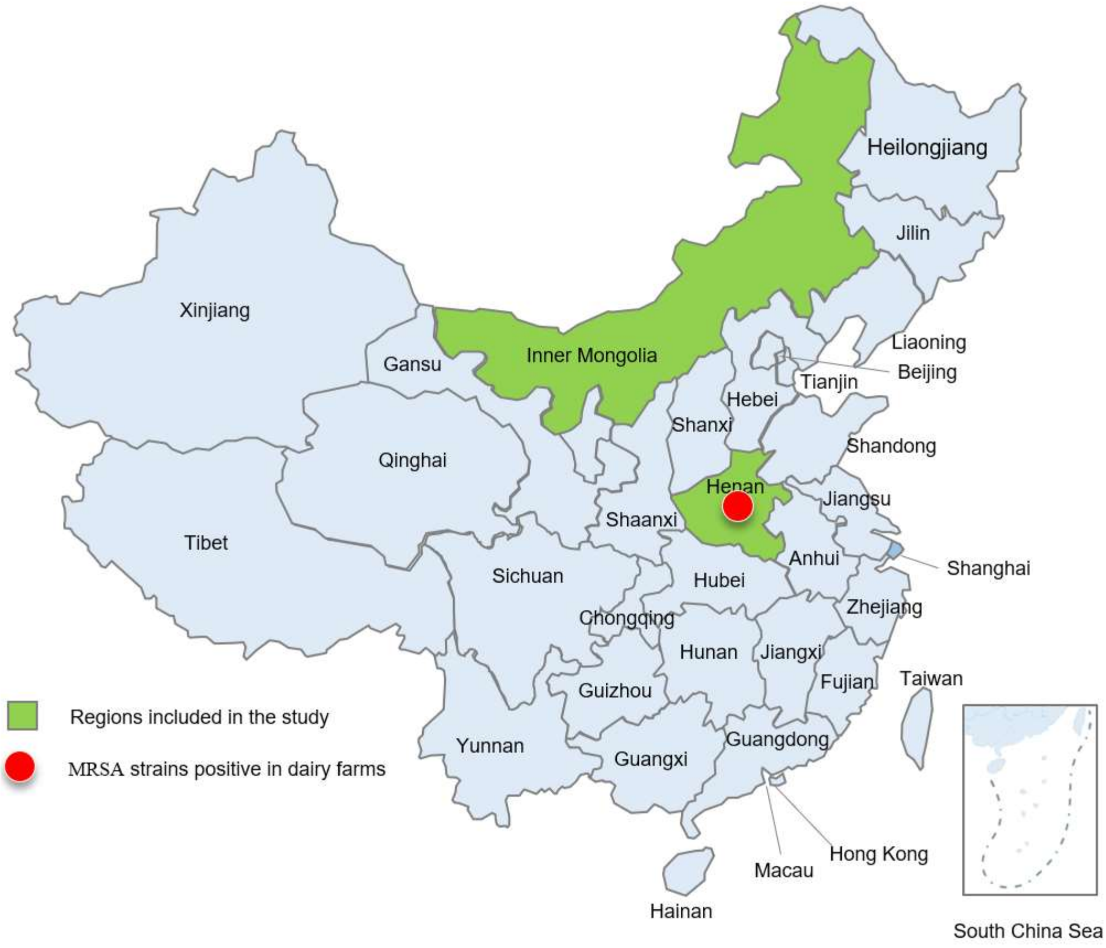

Figure S1. Geographical location of dairy farms presenting *S. aureus* and MRSA isolates in Henan province (HN) and Inner Mongolia autonomous region (IM), China

Table S1. Comparison of the prevalence of raw milk derived *S. aureus* and MRSA in different regions

The resource of *S. aureus* isolates	The number of milk samples	*S. aureus* isolates (%)	MRSA isolates (%)
Henan[a]	122	74 (60.7)	2 (2.7)
Inner Mongolia[a]	33	16 (48.5)	0
Guangdong[b]	80	24 (30.0)	21 (26.3)
Hunan[b]	150	5 (3.3)	3 (2.0)
Zhejiang[b]	224	15 (6.7)	2 (0.9)
Sichuan[b]	249	60 (24.10)	13 (5.2)
Shanghai[b]	150	79 (52.3)	10 (6.7)
Hainan[b]	25	6 (24.0)	0
Jilin[b]	180	24 (13.3)	0
Heilongjiang[b]	100	28 (28.0)	0
Liaoning[b]	100	28 (28.0)	0
Gansu[b]	100	25 (25.0)	0
Jiangsu[c]	1062	117 (11.0)	4 (3.4)
Xinjiang[d]	60	37 (61.7)	14 (37.8)
Bangladesh[e]	47	12 (25.5)	0
Japan[f]	436	135 (31.0)	0

[a] The isolation rate data was presented in this study.

[b, c,] and [d] The isolation rate data was collected from reference 8, 7, and 22, respectively.

[e] and [f] The isolation rate data was collected from reference 20 and 21.
